# Antibiotic Resistance Characterization of Environmental *E. coli* Isolated from River Mula-Mutha, Pune District, India

**DOI:** 10.3390/ijerph15061247

**Published:** 2018-06-12

**Authors:** Rutuja Dhawde, Ragini Macaden, Dhananjaya Saranath, Kayzad Nilgiriwala, Appasaheb Ghadge, Tannaz Birdi

**Affiliations:** 1 The Foundation for Medical Research, 84A, R.G. Thadani Marg, Worli, Mumbai 400 018, India; rutuja713@gmail.com (R.D.); kayzadnilgiriwala@gmail.com (K.N.); 2 St Johns Research Institute, 100 Feet Rd, John Nagar, Koramangala, Bangalore 560 034, India; ragini.macaden@gmail.com; 3 Cancer Patients Aid Association (CPAA), Sumer Kendra, Mumbai 400 0018, India; dhananjaya.saranath@cancer.org.in; 4 The Foundation for Research in Community Health, Pune 411007, India; frchpune@bsnl.in

**Keywords:** Mula-Mutha river, antibiotic susceptibility testing, antibiotic-resistant genes, integrons

## Abstract

In the current study, ceftazidime- and ciprofloxacin-resistant—or dual drug-resistant (DDR)—*E. coli* were isolated from river Mula-Mutha, which flows through rural Pune district and Pune city. The DDR *E. coli* were further examined for antibiotic resistance to six additional antibiotics. The study also included detection of genes responsible for ceftazidime and ciprofloxacin resistance and vectors for horizontal gene transfer. Twenty-eight percent of the identified DDR *E. coli* were resistant to more than six antibiotics, with 12% being resistant to all eight antibiotics tested. Quinolone resistance was determined through the detection of *qnrA*, *qnrB*, *qnrS* and *oqxA* genes, whereas cephalosporin resistance was confirmed through detection of TEM, CTX-M-15, CTX-M-27 and SHV genes. Out of 219 DDR *E. coli*, 8.2% were *qnrS* positive and 0.4% were *qnrB* positive. Percentage of isolates positive for the TEM, CTX-M-15 and CTX-M-27 genes were 32%, 46% and 0.9%, respectively. None of the DDR *E. coli* tested carried the *qnrA*, SHV and *oqxA* genes. Percentage of DDR *E. coli* carrying Class 1 and 2 integrons (mobile genetic elements) were 47% and 8%, respectively. The results showed that antibiotic resistance genes (ARGs) and integrons were present in the *E. coli* isolated from the river at points adjoining and downstream of Pune city.

## 1. Introduction

Antibiotics are required in the treatment of various infectious diseases, namely, diarrhea, pneumonia, urinary tract infections, malaria, tuberculosis and HIV/AIDS [1,2,3,4,5]. However, the present global epidemic of antibiotic-resistant bacteria poses a serious health hazard [6,7,8]. Various studies have been carried out worldwide to estimate actual global burden of antimicrobial resistance [9]. In Europe, antibiotic-resistant bacteria are responsible for the deaths of more than 25,000 patients annually and costs at least €1.5 billion [10]. In the United States, 63,000 deaths occur annually due to antibiotic-resistant bacteria and costs the US health system from 21 to 34 billion dollars annually [9]. Additionally, antibiotic groups, such as cephalosporins and fluoroquinolone, are commonly used worldwide in agriculture practices and in poultry as growth promoters at subtherapeutic doses and to prevent and treat infections [11,12].

In the Indian public health sector, 50% of family spending is associated with unnecessary medications, especially antibiotic treatment [13,14,15]. Nontherapeutic use of antibiotics is also common in the Indian subcontinent, as it is recorded in apiary, poultry, agriculture and aquaculture [16]. Traces of ciprofloxacin, chloramphenicol and erythromycin were detected in branded honey [16]. Colistin, a last resort of antibiotic, was used as a growth promoter in a poultry farm located near Hyderabad city [17]. Antibiotics have also been added to fish and crustacean sea foods in Tamilnadu state prior to export [16]. This increased and indiscriminate use of antibiotics for treatment, in animal husbandry, aquaculture and food preservation in the last two decades has contributed to the growing pool of antibiotic-resistant bacteria [9,18].

The World Health Organization (WHO) endorsed a global action plan in May 2015 to circumvent antimicrobial resistance. This plan is mainly focused on increasing awareness, understanding, surveillance and research on antibiotic resistance towards optimizing antibiotic use, and investment in new drugs, diagnostic tools and vaccines [19]. Government of India has also initiated a 5-year National Action Plan (2017–2021) to combat antibiotic resistance. Under the National Action Plan, Indian Council of Medical Research (ICMR) has set up National Anti-Microbial Resistance Research and Surveillance Network (AMRRSN) to compile national antimicrobial resistance (AMR) data. AMRRSN is focusing on surveillance of diarrheagenic bacterial pathogens, enteric pathogens, enteric organism causing sepsis, Gram-negative non-fermenters and Gram-positive methicillin-resistant *Staphylococcus aureus* [20]. 

The pathogens selected by AMRRSN are not only clinically important but also play a crucial role in the environment by disseminating antibiotic resistance. These pathogens enter fresh and marine waterbodies through sewage disposal [21,22]. The environment plays a critical role in the generation of antibiotic resistance in bacteria. Residual antibiotics in wastewater as pollutants can exert selective pressure and contribute to the increase in antibiotic-resistant bacteria [23]. Sewage from hospitals and the community, agricultural effluent and aquaculture wastewater are also important sources of resistant bacteria polluting the water sources [23,24,25].

Increasing load of antibiotic-resistant bacteria in Indian rivers is a major health concern. Added to this is the inefficient/inadequate sewage treatment, resulting in improperly treated waste being released into rivers [4,15]. Multidrug-resistant bacteria in water sources is a recognized health hazard to the community [26]. Multidrug-resistant bacteria have been isolated from major Indian rivers, including Ganges, Yamuna and Cauvery [27,28,29].

Mula-Mutha is a major river in Pune district in Maharashtra state of India and passes through the center of Pune city. It is a confluence of two rivers, namely, the Mula and Mutha rivers. These two rivers merge at Sangam wadi village, flow through different regions of Pune district and, finally, merge with the river Bhima. It is a monsoon-based river which usually gets its water during the monsoon and dries up in summer. It receives waste from agricultural runoff, disposal of waste, burning of fossil fuels and domestic, hospital and industrial effluents (from small- and large-scale industries) located along the banks of the river [30].

Various new and emerging antibiotic resistance genes (ARGs) have been reported in bacteria isolated from the environment [31]. Recent metagenomic studies revealed that ARGs that cluster in soil and wastewater treatment plants (WWTPs) differ significantly from the ARGs of human pathogens [32,33,34]. Acquisition of resistance genes by bacteria in the environment occurs primarily through horizontal gene transfer. Horizontal gene transfer is facilitated through a variety of mobile gene elements (MGEs), such as plasmids, bacteriophages, genomic islands (GIs), integrative and conjugative elements (ICEs), insertion sequences (ISs), transposons (Tns), integrons and miniature inverted repeat transposable elements (MITEs) [35].

*E. coli* from the Enterobacteriaceae family occur as commensals in human and animal gut [36]. Fluoroquinolones and third-generation cephalosporins are the most commonly used antibiotics in many developing countries, resulting in injudicious use of these antibiotics [37]. β-Lactamases are enzymes produced by microorganisms to inactivate the antibiotics containing β-lactam rings [38]. β-Lactamases can be classified in two different ways, namely, molecular and functional classifications [39]. The molecular classification considers the amino acid sequences. Class A, C, D utilize serine for β-lactam hydrolysis and Class B uses metalloenzymes, which require divalent zinc ions as a cofactor for substrate hydrolysis [39]. Based on functional groups, β-lactamases are classified into three groups, namely, Group 1 cephalosporinases; Group 2 penicillinases, extended-spectrum β-lactamases and serine carbapenemases; and Group 3 metallo-β-lactamases [38]. Extended spectrum β-lactamase (ESBL) production in Gram-negative bacteria enables resistance to a wide variety of penicillin and cephalosporin antibiotics. Additionally, some *E. coli* produce New Delhi metallo-β-lactamase (NDM-1) enzyme that makes them resistant to virtually all β-lactams, including carbapenem [40]. In the case of quinolone resistance, both plasmid and chromosomally mediated resistance are common in *E. coli*, while cephalosporin resistance is commonly coded by plasmids [37].

In the present study, river water samples were screened for ciprofloxacin (fluoroquinolone)- and ceftazidime (third-generation cephalosporin)-resistant thermotolerant fecal coliforms (i.e., dual drug-resistant (DDR) TFC). Further, ciprofloxacin- and ceftazidime-resistant *E. coli* were isolated from DDR TFC and were subjected to antibiotic susceptibility testing to determine their antibiotic resistance profile and ESBL production. DDR *E. coli* were also tested for genes coding for β-lactamase production (TEM, SHV, CTX-M-15 and CTX-M-27) and quinolone resistance (*qnrA*, *qnrB*, *qnrS*, *oqxA*) and horizontal gene transfer (HGT) genes—the Class 1 and 2 integrons (*intI1*, *intI2*).

## 2. Materials and Methods 

### 2.1. Study Area and Sample Collection

**Figure 1:**
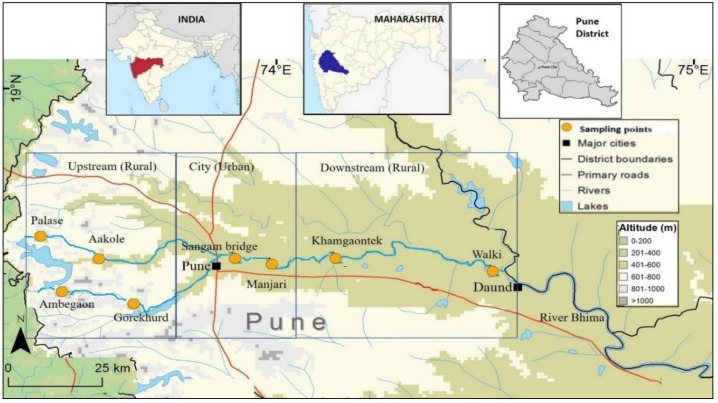
Distribution of sampling points across Mula and Mutha river.

Eight sampling sites along the Mula-Mutha river were selected, covering the whole length of the river including upstream, confluence and downstream locales with respect to Pune city. Of the eight sampling points, six were from rural and two were from urban Pune (Figure 1). The distance between sampling points ranged from 5–40 km. Three water samples per site were collected over a period of 1 year. The samples were collected three times over a period of 1 year: during post-monsoon (January 2016), pre-monsoon (May 2016) and monsoon (August 2016). Approximately 400 mL river water was collected in previously autoclaved 500 mL polypropylene bottles from 60 cm beneath the river surface. These bottles were immediately kept on ice and transported to the laboratory in Mumbai (6–8 h) on the same day, stored overnight between 4–8 °C, and bacteriological analysis was undertaken on the following day [41]. Bacterial analysis of the water samples was carried out as per the specifications given by the Bureau of Indian Standards [42].

### 2.2. Isolation of Dual Drug-Resistant (DDR) E. coli

Selection of antibiotics for the study was based on literature survey [43,44] and information obtained regarding antibiotics prescribed by Public Health Centre (PHC) doctors or private practitioners and over-the-counter antibiotics dispensed by private and PHC chemists in selected villages (data not shown).

To isolate and enumerate dual drug-resistant (DDR) TFC, cellulose acetate filter-sterilized ceftazidime (16 µg/mL) and ciprofloxacin (4 µg/mL) were added to membrane fecal coliform (m-FC) agar prior to pouring into Petri dishes. The concentrations of antibiotics used were as per Centre for Clinical and Laboratory Standards Institute guidelines [45]. DDR TFC were enumerated using different volumes of water ranging from 10^−2^ mL to 10^2^ mL filtered through cellulose acetate 0.22 µ filters (Merck Millipore, Darmstadt, Germany). The filter papers were placed face-upward on m-FC agar and incubated at 44.5 °C for 24 h. The filter papers were observed for blue colored colonies and results expressed as colony forming units (CFU) per 100 mL. Additionally, the total TFC was enumerated using the membrane filtration technique and m-FC medium without incorporating antibiotics into the medium. DDR TFC colonies were streaked on Hichrome *E. coli* agar (Himedia, Mumbai, India). Bluish-green colonies on the media represented DDR *E. coli*. Dual drug-resistant *E. coli* were preserved in 15% glycerol stocks in sterile Luria Bertani broth and stored at −20 °C [45]. Cell density of DDR *E. coli* in the glycerol stock was maintained as 10^8^ cells/mL.

### 2.3. Antibiotic Susceptibility Testing

To revive DDR *E. coli* from frozen glycerol stock, 0.1 mL of stock was added to 1 mL Luria Bertani broth (Himedia, Mumbai, India) and incubated overnight at 37 °C with aeration. The resultant actively growing DDR *E. coli* culture was centrifuged at 6797× *g* for 10 min in an Eppendorf centrifuge 5430 (Hamburg, Germany). The supernatant was decanted. Cells were washed with saline and centrifuged again at 6797× *g* for 10 min followed by decanting of the supernatant. Saline was added to the DDR *E. coli* pellet followed by cell density adjustment to 10^8^ cells/mL using MacFarland standard of 0.5 (which is equal to a cell density 1.5 × 10^8^ CFU/mL) (Himedia, Mumbai, India). After adjusting the cell density, DDR *E. coli* were subjected to antibiotic susceptibility test (AST) by disk diffusion method on Mueller Hinton agar plates (Himedia, Mumbai, India) [46]. *E. coli* ATCC25922—a pan-sensitive strain—served as a negative control [47]. Hexadisc G15 minus (Himedia, Mumbai, India) containing a panel of six antibiotics was used for susceptibility testing. The six antibiotics in the Hexadisc were ampicillin (10 µg), cefepime (30 µg), cefotaxime (30 µg), gentamicin (10 µg), imipenem (10 µg) and piperacillin/tazobactam (10/10 µg). Antibiotic resistance was estimated by measuring the respective zones of inhibition (diameters) around each antibiotic disk as per Clinical and Laboratory Standards Institute [45]. The presence of ESBLs was confirmed by double-disk synergy test using ceftazidime (30 µg) and a combination of ceftazidime (30 µg) and clavulanic acid (10 µg) [48].

### 2.4. Genetic Determinants of Antibiotic Resistance and Horizontal Gene Transfer (HGT) in DDR E. coli (HGT)

Glycerol stock of DDR *E. coli* was revived in Luria Bertani broth. Overnight culture of DDR *E. coli* was centrifuged at 6797× *g* for 10 min followed by DNA extraction using PureLink® Genomic DNA Kit (Invitrogen, CA, USA). The DNA was subjected to agarose gel electrophoresis (AGE) in 1% agarose gel, quantitated using NanoDrop 2000 (Thermo Fisher Scientific, MA, USA) at 260 and 280 nm and amplified by polymerase chain reaction (PCR) using Master Cycler Gradient (Eppendorf, Hamburg, Germany). Specific primers to TEM, SHV, CTX-M-15, CTX-M-27, *qnrA*, *qnrB*, *qnrS*, *intI1*, *intI2*, *oqxA* genes were purchased from Sigma Aldrich (MO, USA) (sequences detailed in Table 1). PCR master mix was purchased from Bioron (Ludwigshafen, Germany). The PCR was carried out in a final volume of 25 µL. Absence of PCR inhibitors in the sample DNAs was observed by PCR amplification of at least one gene of investigation in the majority (86%) of the extracted DNA from the identified isolates.

In the present study, TEM, CTX-M-15, CTX-M-27 were multiplexed as Group I; *qnrA*, *qnrB* and *qnrS* formed multiplexed as Group II; *oqxA*, SHV were multiplexed as Group III; and *intI1* and *intI2* were amplified individually. The PCR conditions used for multiplexes and single-plex were as follows: initial denaturation at 95 °C for 120 s, followed by 32 cycles of amplification at 95 °C for 45 s, annealing at different temperatures as mentioned in Table 1. The temperature for extension was 72 °C for 60 s. The final extension was at 72 °C for 5 min. The extension for integron PCRs was carried out for 68 °C for 60 s. PCR products were electrophoresed on 1.5% (*w*/*v*) agarose gel and stained with ethidium bromide. Additionally, representative positive PCR products were subjected to Sanger sequencing through an ABi 3730Xl sequencer (Applied Biosystem, CA, USA). DDR *E. coli* harboring different ARGs and mobile genetic elements were enumerated. 

Correlation between HGT and β-lactamase or fluoroquinolone resistance was analyzed by statistical χ^2^-test.

PCR Conditions were as follow.


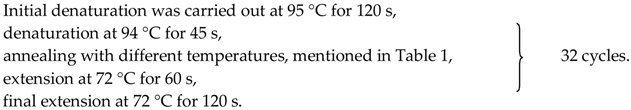


Note: In the case of integron PCR, extension was carried out for 68 °C for 60 s.

## 3. Results

### 3.1. Antibiotic Resistance Profile of DDR E. coli

The ratio of DDR *E. coli* to TFC was calculated and expressed in percentages across different sampling sites (Table 2). The average load of DDR *E. coli* was found to be 4.3 × 10^4^ CFU/100 mL in Mula-Mutha river over a period of 1 year, and 219 isolates were tested for phenotypic and genotypic resistance.

DDR *E. coli* were not detected in the river upstream (Palase, Aakole, Ambegaon, Gorekhurd) and extreme downstream (Walki) of Pune city. The load was concentrated at Sangam bridge, Manjari and Khamgaontek, which constitute confluence of Mula and Mutha rivers, in the city and downstream of city (Table 2). Percentages of DDR *E. coli* showing resistance at points adjoining and downstream of Pune city to various antibiotics and ESBL production are detailed in Table 2 and Table 3. It was found that 63% were resistant to more than six antibiotics, with 28% being resistant to all the eight antibiotics tested (Table 3). All DDR *E. coli* showed resistance to ampicillin. Ninety-eight percent of the isolates were resistant to cefotaxime, which is a third-generation cephalosporin-like ceftazidime. Resistance to cefepime (fourth-generation cephalosporin) and piperacillin/tazobactam combination was 75% and 73%, respectively. Forty-five percent of DDR *E. coli* were resistant to imipenem, which is considered as a “reserve “antibiotic. Resistance to gentamicin was noted in 33% of the isolates (Table 2). Only 10% of the isolates were ESBL producers (Table 2). 

### 3.2. Detection of Genes Responsible for Fluoroquinolone and Cephalosporin Resistance in DDR E. coli

Fluoroquinolone: All isolates were examined for the presence of plasmid-mediated genes *qnrA*, *B*, *S* and efflux pump-mediated gene *oqxA.* A single isolate from total of 219 tested harbored *qnrB* and 18 isolates harbored the *qnrS* gene. No isolate contained either *qnrA* or *oqxA* genes. 

Cephalosporin: The DDR *E. coli* harbored CTX-M-15 (46%) and TEM (32%), and 10% of the isolates harbored both TEM and CTX-M-15. Two isolates carried both the genes CTX-M-27 and TEM. None of the isolates harbored SHV (Table 4). 

Although the 219 DDR *E. coli* were initially isolated as colonies resistant to ceftazidime and ciprofloxacin, only 4.5% of the isolates harbored resistant genes for both antibiotics (TEM and *qnrS*).

### 3.3. Horizontal Gene Transfer (HGT) of Antibiotic Resistance through Integron 1 and 2 in DDR E. coli

Forty-seven percent of the DDR *E. coli* isolates carried the *intI1* gene, whereas 8% carried the *intI2* gene. Only 2% of the isolates carried both *intI1* and *intI2* genes (Table 4). 

Fluoroquinolone resistance was not significantly associated with HGT (χ^2^ == 0.203, df = 1, α > 0.05), whereas β-lactam resistance was significantly associated with HGT (χ^2^ == 1.194, df = 1, α < 0.001). No isolate carried genes for β-lactamase production, quinolone resistance and HGT together (Table 4).

## 4. Discussion

Pradhan (2016) investigated the river Mula-Mutha for different types of pollution [30] and highlighted the role of malfunctioning of Pune wastewater treatment plants (WWTPs) and their role in the deterioration of the Mula-Mutha’s water quality. Pune Municipal Corporation (PMC) has installed 10 WWTPs in the city for treatment of 570 million liters of wastewater per day (MLD) from the city. However, only 290 MLD of the city’s waste water is actually treated and 50% of the untreated sewage is released into the river [30]. These observations corroborate our findings of fecal contamination of the river water at points adjoining and downstream of the city.

### 4.1. Antibiotic Resistance Profile of DDR E. coli

A scoping report on “Antimicrobial Resistance in India” published by Department of Biotechnology, New Delhi, India in 2017 highlighted increasing load of antibiotic-resistant bacteria and ARGs in Indian surface waterbodies and major drinking water sources [54].Various other studies have also been undertaken across India to check for the presence of antibiotic-resistant bacteria in surface and groundwater sources [55,56]. These studies highlighted that the majority of rivers flowing in Bihar, Goa, Karnataka, Tamilnadu, Telangana states are contaminated with multidrug-resistant *E. coli*. Additionally, major drinking water sources from villages situated along the banks of river Sharayu, which flows through Uttar Pradesh and Uttarakhand states, are also contaminated with drug-resistant bacteria [54]. Our findings on Mula-Mutha reflect the situation in other Indian rivers, since a large proportion of the TFC isolated in this study were multidrug resistant. The heavy load of AR TFC detected in the river at points adjoining and downstream of Pune city could be explained by the observations of Keche et al., who reported that antibiotics cefexime, ciprofloxacin and tinidazole in combination and the amoxicillin–clavulanic acid combination are commonly prescribed in Pune city [43]. Additionally, they reported that, at times, antibiotics were sold on outdated prescriptions by physicians (64.32%), thus contributing to the injudicious use, and, at other times, antibiotics were taken based on a chemist’s or neighbor’s advice, resulting in overuse/inappropriate use (23%). Another study stated that self-medication with antibiotics was commonly observed in rural Pune. All these practices could explain the increasing antibiotic resistance in the bacteria in the study area [44]. 

All the isolated DDR *E. coli* were resistant to ampicillin (penicillin) and cefotaxime (third-generation cephalosporin) (Table 2). Similarly, Skariyachan et al. (2015) isolated fecal bacteria from the river Cauvery which were resistant to multiple antibiotics, including penicillin and third-generation cephalosporins [29]. We also observed that a greater number of DDR *E. coli* were resistant to imipenem than to gentamicin (Table 3). Odenholt et al. (1989) opined that use of antimicrobials, such as the polymyxins, fosfomycin and gentamicin, was infrequent due to lesser therapeutic efficacy and/or toxicity. The bacterial population was not, therefore, exposed to these antimicrobials, accounting for higher susceptibility of the bacteria to gentamicin [57]. Similar to our findings, increasing numbers of carbapenem-resistant Enterobacteriaceae bacteria have been recorded worldwide [58]. A report by Centers for Disease Control and Prevention stated that, in United States, 140,000 patients get affected annually with bacteria from the Enterobacteriaceae family, out of which 9300 are Carbapenem-Resistant Enterobacteriaceae (CRE), and 600 deaths annually are attributed to these CRE [58].

An interesting observation in the present study was the minimal burden of antibiotic-resistant TFC and DDR *E. coli* at Walki. The probable reasons could be the presence of bacteriophages in the river [59], or a dilution of river water due to backflow of river Bhima into Mula-Mutha [60,61]

### 4.2. Genotypic Antibiotic Resistance in DDR E. coli

In the present study, plasmid-mediated cephalosporin and β-lactam resistance in DDR *E. coli* was studied through detection of TEM, CTX-M-15, CTX-M-27 genes. The percentage DDR *E. coli* harboring CTX-M-15 was 46%, and TEM was 32% (Table 4). In accordance with the current study, Akiba et al. (2015) reported that 66% *E. coli* from various Indian rivers harbored the CTX-M group of genes, predominantly CTX-M-15 (44%) and TEM (44%) [24]. Generally, TEM and CTX-M genes coexist on plasmids [62]. The isolated DDR *E. coli* lacked SHV genes, as SHV is mainly reported in *Klebsiella* species [63]. On the other hand, ESBLs are generally derived from TEM1, TEM2 or SHV1 mutated genes, although genes other than the TEM or SHV lineage may be responsible for an increasing number of ESBLs. The data by Virdi and Singh (2017) [64] confirms our results of fewer DDR *E. coli* producing ESBL, despite 32% of the isolates harboring TEM.

In the present study, 73% of the isolates showed resistance to the piperacillin–tazobactam (β-lactam + inhibitor) combination (Table 2). Resistance to these antibiotics is primarily chromosome mediated [49]. The emphasis of the current study was on plasmids and integrons and did not include detection of the chromosomal genes coding for resistance to piperacillin–tazobactam. 

In the current study, *qnrA*, *B* and *S* genes were chosen to investigate plasmid-mediated quinolone resistance (PMQR). Although the isolated DDR *E. coli* strains were resistant to ciprofloxacin, only 8% of DDR *E. coli* carried the *qnrS* gene (Table 4). Singh and Virdi (2017) detected the PMQR gene *qnrS* in 15% of *E. coli* isolated from river Yamuna [64]. Fluoroquinolone resistance determinant *qnrA* was absent, whereas a single DDR *E. coli* harbored *qnrB* (Table 4). The absence of *qnrA* and *qnrB* could be due to the genes being often embedded in complex sul1-type integrons [65]. These structures commonly occur in integron Class 6 and Class 7 [21], which were not included in the present study. Fluoroquinolone resistance could also be mediated through genes encoding efflux pump proteins, such as *oqxAB*, *qepA1* and *qepA2* [66]. The present study was, however, restricted to detection of the *oqxA* gene, which was not found in any DDR *E. coli* strain isolated in the study. The DDR *E. coli* isolated in the current study did not harbor the *oqxA* gene (Table 4). A report by Kim et al. (2009) revealed that *oqxA* and *oqxB* genes are primarily associated with swine manure samples [51]. Porcine farms were not observed along the banks of Mula-Mutha river, which corroborates our finding of lack of efflux pump-driven fluoroquinolone resistance in DDR *E. coli*. Therefore, resistance to fluoroquinolones in 8% of the DDR *E. coli* was attributed to the presence of the plasmid-mediated *qnrS* gene. In the remaining isolates, the resistance may be attributed to chromosomally mediated resistance due to point mutations in the topoisomerase subunits GyrA, GyrB, ParC or ParE (the detection of which was not included in the study) [67].

### 4.3. Horizontal Gene Transfer of Antibiotic Resistance in DDR E. coli

Integrons carrying gene cassettes for multidrug resistance are important in the development of antibiotic resistance in Gram-negative bacteria [68]. In the current study, horizontal transfer was studied through detection of *intI1* and *intI2*. The load of *intI1* was 47% and *intI2* was 18% (Table 4). In an earlier study by Sunde and colleagues, the prevalence rate of integrons ranged from 22% to 59% in the Enterobacteriaceae family [69]. Similarly, a retrospective surveillance for integrons conducted in China during 2001–2005 detected 5.7% *intI2* in a variety of species, including *Pseudomonas aeruginosa*, *Escherichia coli*, *Enterobacter faecalis*, *Proteus vulgaris* and *Proteus mirabilis* [70]. 

The frequency of co-existence of *intI1* and *intI2* in *E. coli* in the present study was 2% (Table 4). A similar observation was made by Kortlaska et al. (2015), who reported both classes of integrons in only one *E. coli* isolate (0.38%) [63].

A recently published study by Marathe et al. reported similar findings of detection of ARGs and mobile genetic elements in the river Mutha [71]. Marathe et al. detected horizontally transferable ARGs, including carbapenemases, namely, NDM, VIM, KPC, OXA-48 and IMP types, loaded in the river Mutha using the shotgun sequencing method [71].

In the current study, a high load of DDR *E. coli* carrying *intI1* was detected in the river at Pune city and downstream of the city. This suggests that the WWTPs located in the city may be major contributors of ARGs and integrons released in rivers. Wastewater treatment plants receive sewage with dense and diverse microbes and treats it through primary, secondary and tertiary treatments [72]. Activated sludge, which constitutes secondary treatment, may facilitate horizontal transfer of ARGs in WWTPs [73]. Aubertheau et al. [74] has explained the role of WWTPs in antibiotic resistance dissemination in Vienne River, France. Lapara et al. (2001) has also mentioned the role of municipal WWTPs as a source of ARGs and mobile genetic elements in Duluth-Superior Harbor [75]. Class 1 integrons are prevalent in Gram-negative bacteria, including *Pseudomonas*, *Salmonella*, *Shigella*, *Escherichia* [76]. The rate of HGT is high in prokaryotes and can lead to transfer within and between bacterial species [77,78,79]. Furthermore, *intI1* genes are also accompanied by the genes conferring resistance to heavy metals and disinfectants [80]. This suggests that contamination of the river with chemicals and heavy metals near urban areas can serve as selective pressures in the environment to generate antibiotic-resistant bacteria. 

Mula-Mutha river water is used for drinking after preliminary treatment, like chlorination, and, at times, after a coarse filtration through cloth in villages. The river water is also used for bathing animals, cleaning utensils and for irrigation in several villages downstream of Pune city. Antibiotic-resistant *E. coli* present in water are capable of causing infections, such as diarrhea and wound infections. Treating these infections poses a problem because the bacilli are multidrug resistant. Moreover, the *E. coli* serve as reservoirs of plasmids carrying genes coding for antibiotic resistance which can be transferred horizontally, aided by the presence of integrons, to other bacteria, and spread antibiotic-resistant bacteria in the environment.

## 5. Conclusions

Increasing numbers of antibiotic-resistant bacteria in the river represent a cumulative effect of exponentially rising population in Pune city, overburden on WWTPs, resulting in inefficient functioning, poor sanitation and irrational use of antibiotics. Around 50% of DDR *E. coli* isolated from Mula-Mutha harbored genes which protect bacteria against “reserve” drugs, including imipenem, and are capable of propagating horizontal gene transfer. Results of the present study emphasize the need to undertake corrective interventions at the national and state level. Compliance with the WHO Antibiotic Stewardship Program (ASP) in public health would improve the treatment of infections and curtail adverse effects associated with antibiotic use [81]. Additionally, measures should be taken at Municipal Corporation and village Panchayat levels to improve sanitation and protect rivers from sewage and other harmful wastes generated from the surrounding area. It is necessary to invest in efficient WWTPs in cities and in villages to decontaminate wastewater before it flows into the river or other water sources. In addition, place strong and effective legislations/laws are needed to control, regulate and prevent sale of antibiotics without prescriptions from qualified medical practitioners. These measures, together with creating awareness in the community on the dangers of antibiotic-resistant bacteria spreading in the environment, will go a long way to control the problem.

## Figures and Tables

**Table 1:** Sequences of primers, PCR conditions, annealing temperature for amplifying target genes.

Description	Target Genes	Sequence (5′-3′)	Amplicon Size	Annealing Temp (°C)	Reference
β-Lactamase	SHV	FP: AGCCGCTTGAGCAAATTAAAC RP: ATCCCGCAGATAAATCACCAC	713	64	[49]
	TEM	FP: CATTTCCGTGTCGCCCTTATTC RP: CGTTCATCCATAGTTGCCTGAC	800	62	
	CTX-M-15	FP: TTAGGAARTGTGCCGCTGYA RP: CGATATCGTTGGTGGTRCCAT	688		
	CTX-M-27	FP: TCAAGCCTGCCGATCTGGT RP: TGATTCTCGCCGCTGAAG	561		
Plasmid-mediated quinolone resistance	*qnrA*	FP: AAGGAAGCCGTATGGATATT RP: AGCTAATCCGGCAGCACTAT	670	54	[50]
	*qnrB*	FP: CGACCTGAGCGGCACTGAAT RP: TGAGCAACGATGCCTGGTAG	515		
	*qnrS*	FP: ACCTTCACCGCTTGCACATT RP: CCAGTGCTTCGAGAATCAGT	571		
	*oqxA*	FP: CTCGGCGCGATGATGCT RP: CCACTCTTCACGGGAGACGA	280	64	[51]
Horizontal gene transfer	*intI1*	FP: CCTCCCGCACGATGATC RP: TCCACGCATCGTCAGGC3	280	55	[52]
	*intI2*	FP: CACGGATATGCGACAAAAAGGT RP: GTAGCAAACGAGTGACGAAATG	788	55	[53]

**Table 2:** Dual drug-resistant (DDR) *E. coli* load at different sites and their antibiotic resistance profile.

	DDR *E. coli*—CP and CZ Resistant (%)									
Sampling Site	CFU (Log_10_)/100 mL [%]	Resistance to								
		Fluro-Quinolone	Cephalosporin			Penicillin	Carbapenem	Amino Glycoside	β-Lactam and Inhibitor	ESBL Production
		CP	CZ	CTX	CPM	AMP	IMP	GN	PIT	CZ + CZL
			(3rd gen)	(3rd gen)	(4th gen)					
KT	1.99 [2.2]	94	94	94 (100)	75 (79)	94 (100)	44 (46)	16 (17)	71 (75)	6 (6)
MJ	3.61 [1.2]	60	60	57 (92)	41 (68)	60 (100)	26 (43)	5 (8)	33 (55)	6 (10)
SB	5.09 [1.8]	65	65	64 (98)	49 (75)	65 (100)	29 (44)	12 (18)	56 (86)	10 (15)
Total			219	215 (98)	165 (75)	219 (100)	99 (45)	33 (15)	160 (73)	22 (10)

Abbreviations: KT: Khamgaontek, MJ: Manjari, SB: Sangam Bridge, CFU: Colony forming units, EBSL: Extended spectrum β-lactamase, CP: Ciprofloxacin, CZ: Cephatazidime, CTX: Cefotaxime, CPM: Cefepime, AMP: Ampicillin, IMP: Imipenem, GN: Gentamicin, PIT: Piperacillin with Tazobactam.

**Table 3:** Extended antibiotic resistance profile of DDR *E. coli.*

Resistance to Number of Antibiotics	Ciprofloxacin and Ceftazidime (CZ) + Additional Antibiotics	Resistant Isolates (%)
3	AMP/CPM/CTX/PIT	4 (1.8)
4	CTX and AMP/IMP/GN/PIT/CPM CPM and AMP/IMP/GN/PIM GN and IMP/PIT IMP and PIT	22 (10)
5	CTX + CPM and AMP/IMP/GN/PIT CTX + AMP and IMP/GN/PIT CTX + IMP and GN/PIT CTX + IMP and GN/PIT CPM + AMP and IMP/GN/PIT AMP + GN and PIT IMP + GN and PIT	39 (17.8)
6	CTX + CPM + AMP and IMP/PIT/GN CTX + AMP + IMP and GN/PIT/CPM AMP + IMP + GN and PIT/CPM/CTX AMP + GN + PIT and CTX AMP + PIT+ CTX and CPM IMP + GN + PIT and CTX IMP + PIT+ CTX and CPM IMP + CTX + CPM and AMP GN + PIT + CTX and CPM GN + CTX + CPM and AMP GN + CPM + AMP and IMP	63 (28.7)
7	CTX + CPM + AMP + IMP and GN/PIT CTX + CPM + GN + PIT and AMP/IMP	63 (28.7)
8	CP + CZ+ CTX + CPM + AMP + IMP + GN + PIT	28 (12.7)

Abbreviations: CP: Ciprofloxacin, CZ: Cephatazidime, CTX: Cefotaxime, CPM: Cefepime, AMP: Ampicillin, IMP: Imipenem, GN: Gentamicin, PIT: Piperacillin with Tazobactam.

**Table 4:** DDR *E. coli* (%) detected with specific resistance and/or with integrons.

Genes	No. of DDR *E. coli* (%)
Associated with fluoroquinolone	
*qnrB*	1 (0.4)
*qnrS*	18 (8.2)
Associated with β-lactamase	
TEM	70 (32)
CTX-M-15	100 (46)
CTX-M-27	2 (0.9)
TEM + CTX-M-15	22 (10)
TEM + CTX-M-27	2 (0.9)
Associated with fluoroquinolone + β-lactamase	
*qnrS +* TEM	10 (4.5)
Associated with HGT	
*intI1*	103 (47)
*intI2*	18 (8)
*intI1 + intI2*	5 (2)
Associated with fluoroquinolone + Class 1 integron	
*qnrB + intI1*	1 (0.4)
*qnrS + intI1*	10 (4.5)
Associated with fluoroquinolone + Class 2 integron	
*qnrS+int2*	1 (0.4)
Associated with β-lactamase + Class 1 integron	
CTX-M-15 *+ intI1*	55 (25)
CTX-M-27 *+ intI1*	1 (0.4)
TEM *+ intI1*	72 (32)
TEM + CTX-M-15 *+ intI1*	14 (6)
TEM + CTX-M-27 + *intI1*	1 (0.4)
Associated with β-lactamase + Class 2 integron	
CTX-M-15 *+ intI2*	11 (5)
TEM *+ intI2*	7 (3.1)
TEM + CTX-M-15 *+ intI2*	3 (1.3)
Associated with β-lactamase + Class 1 and Class 2 integron	
CTX-M-15 *+ intI2 + intI1*	3 (1.3)
TEM *+ intI2 + intI1*	4 (1.8)
TEM + CTX-M-15 *+ intI2 + intI1*	2 (0.9)
